# Supplementary material for: Spectrum and Prevalence of Rare APOE Variants and Their Association with Familial Dysbetalipoproteinemia
Source: Int J Mol Sci. 2024 Nov 25;25(23):12651. doi: 10.3390/ijms252312651 (PMC11641494; doi:10.3390/ijms252312651)
Supplement: Supplementary file 1 [file ijms-25-12651-s001.zip › ijms-3275015-supplementary/Figure S1_Supplementary Material.pdf]

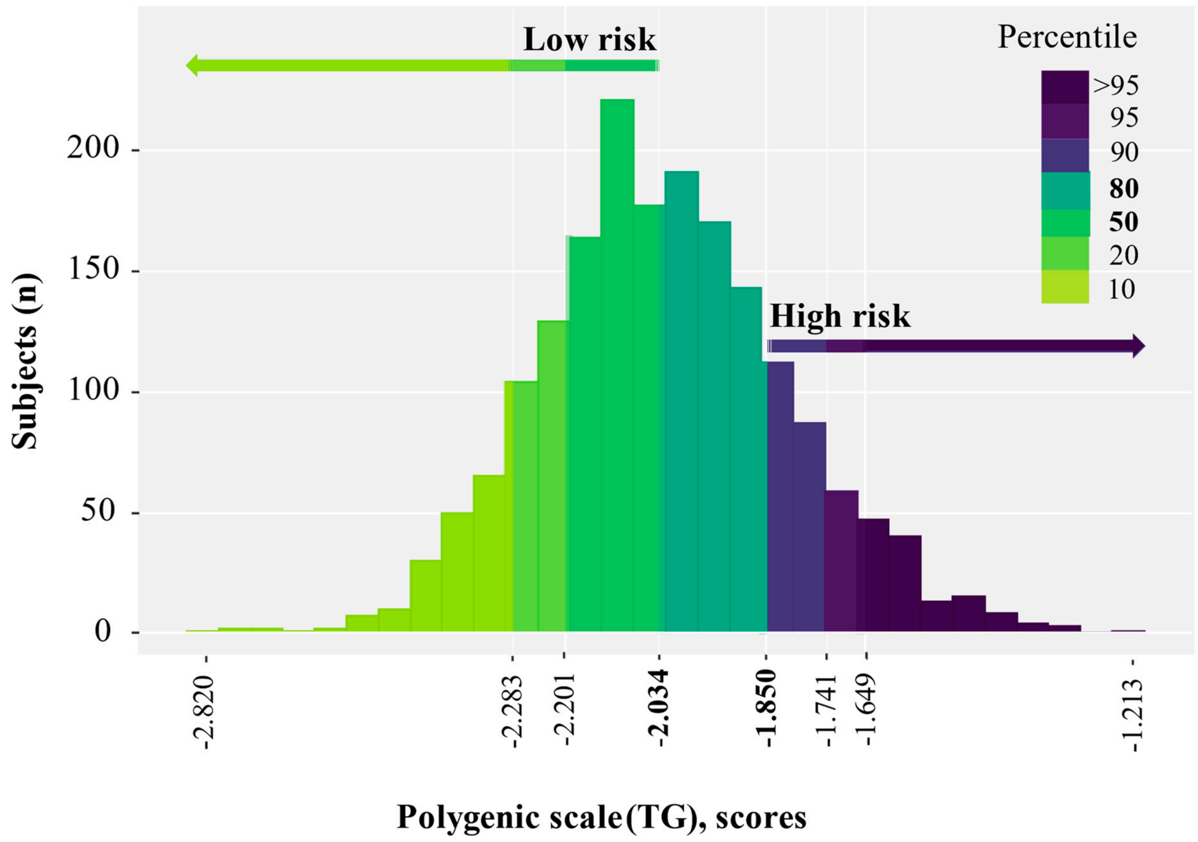

Supplementary Figure S1. Polygenic risk score distribution of TG levels among ESSE-Ivanovo sample (n = 1858). The x-axis shows the distribution of the polygenic risk score, and the y-axis shows the number of subjects. Color indicates percentiles. TG—triglycerides.
