# Supplementary material for: Spectrum and Prevalence of Rare APOE Variants and Their Association with Familial Dysbetalipoproteinemia
Source: Int J Mol Sci. 2024 Nov 25;25(23):12651. doi: 10.3390/ijms252312651 (PMC11641494; doi:10.3390/ijms252312651)
Supplement: Supplementary file 1 [file ijms-25-12651-s001.zip › ijms-3275015-supplementary/Table S1_Supplementary Material.pdf]

Supplementary Table S1. Characteristics of the ESSE-Ivanovo sample.

Parameter	ESSE-Ivanovo sample<br>(*n* = 1858)
Men, n (%)	679 (36.5)
Age, years, Me (Q1; Q3)	48 (37; 56)
BMI, kg/m^2, Me (Q1; Q3)	27.9 (24.4; 31.7)
Diabetes, n (%)	91 (4.9)
Hypertension, n (%)	1355 (72.9)
Coronary heart disease, n (%)	80 (4.3)
Statins, n (%)	94 (5.1)<br>*n* = 1849[1]
Total cholesterol, mmol/L, Me (Q1; Q3)	5.43 (4.69; 6.26)
LDL-C, mmol/L, Me (Q1; Q3)	3.18 (2.41; 4.00)
HDL-C, mmol/L, Me (Q1; Q3)	1.39 (1.19; 1.63)
TG, mmol/L, Me (Q1; Q3)	1.21 (0.85; 1.79)
Lp(a), mg/dL, Me (Q1; Q3)	9.5 (4.8; 27.2)

[1]Nine subjects had no data on lipid-lowering therapy. BMI—body mass index; HDL-C—high-density lipoprotein cholesterol; LDL-C—low-density lipoprotein cholesterol; Lp(a)—lipoprotein (a); Me—median; TG—triglycerides.
